# The relationship between perfectionism, neuroticism, and exercise addiction risk: a latent profile analysis and the mediating role of social physique anxiety

**DOI:** 10.3389/fpsyg.2026.1857926

**Published:** 2026-06-26

**Authors:** Junhao Weng, Chen Tang, Linbo Sha

**Affiliations:** School of Physical Education, Henan University, Kaifeng, China

**Keywords:** exercise addiction, latent profile analysis, neuroticism, perfectionism, social physical anxiety

## Abstract

**Background:**

Exercise addiction (EA) is an emerging public health concern among university students, yet limited attention has been paid to heterogeneity in personality-related risk profiles. This study aimed to identify latent profiles based on perfectionism and neuroticism and to examine whether social physique anxiety (SPA) statistically mediates the association between these profiles and EA.

**Methods:**

Between April and May 2025, this study surveyed 481 university students using the Frost Multidimensional Perfectionism Scale (FMPS), the NEO Five-Factor Inventory-Neuroticism Subscale (NEO-FFI-N), the Social Physique Anxiety Scale (SPAS), and the Exercise Addiction Inventory (EAI). Data from 481 participants were processed using SPSS 26.0. Latent profile analysis was conducted using Mplus 8.0, and mediation analysis was performed using the PROCESS 4.1 macro.

**Results:**

Latent profile analysis identified three perfectionism–neuroticism profiles: low (32.64%), moderate (57.38%), and high (9.98%). Compared with the low perfectionism–neuroticism profile, both the moderate and high profiles were associated with higher EAI scores. The mediation analysis was consistent with a partial statistical mediating role of SPA in these associations. For the moderate profile, the total effect on EAI scores was 0.78 (SE = 0.10, 95% CI [0.59, 0.97]), the direct effect was 0.54 (SE = 0.10, 95% CI [0.35, 0.73]), and the indirect effect through SPA was 0.24 (SE = 0.04, 95% CI [0.16, 0.34]). For the high profile, the total effect on EAI scores was 1.60 (SE = 0.16, 95% CI [1.28, 1.92]), the direct effect was 1.18 (SE = 0.16, 95% CI [0.86, 1.50]), and the indirect effect through SPA was 0.43 (SE = 0.08, 95% CI [0.28, 0.60]).

**Conclusion:**

University students with high-risk personality traits showed higher EAI scores, partly through elevated SPA. This psycho-behavioral association highlights the need to move beyond a purely behavior-focused perspective and adopt a more integrated approach that considers both personality traits and social evaluative sensitivity. Enhancing identification and intervention mechanisms for at-risk individuals may help promote more stable and sustainable patterns of physical activity among university populations.

## Introduction

Regular physical activity provides substantial benefits for physical and mental health, including improvements in cognitive health, health-related quality of life, mental health, and sleep quality (Ding et al., 2020; Y. Zhu et al., 2025). However, when exercise becomes excessive, compulsive, and difficult to control, it may develop into a maladaptive behavioral pattern known as exercise addiction (EA) (Berczik et al., 2012). EA is associated with multiple adverse outcomes, including musculoskeletal injuries, negative emotional states, and social impairment, and has therefore become an important issue in exercise psychology and public health (Back et al., 2021; Li and Ren, 2019; Olave et al., 2021). Although existing research has examined EA among fitness enthusiasts and professional athletes (Di Lodovico et al., 2019; Mayolas-Pi et al., 2025; Zeeck et al., 2017), university students also constitute a population requiring attention. Previous studies have reported that a non-negligible proportion of university students may be at risk of EA, particularly among students with regular exercise habits or sports-related academic backgrounds (Griffiths et al., 2005; Hausenblas and Downs, 2002; Szabo and Griffiths, 2007). Given this evidence, the present study focused on university students with regular exercise participation, among whom EA-related risk can be more appropriately assessed.

The interactional model of EA proposed by Egorov and Szabo (2013) suggests that EA may result from the interaction of biological, psychological, and social environmental factors. Based on the interactional model proposed by Egorov and Szabo (2013), Figure 1 presents a redrawn conceptual framework illustrating the biological, psychological, and social factors related to exercise addiction risk (Egorov and Szabo, 2013). From this perspective, EA involves the combined influence of individual vulnerability and social-contextual pressure. However, much of the existing literature has examined psychological predictors of EA using variable-centered approaches, focusing on whether a single trait or psychological factor is linearly associated with EA. Such approaches are useful for identifying general risk factors, but they provide limited insight into whether different configurations of personality traits exist within individuals and whether these configurations are differentially associated with EA. Therefore, a more person-centered perspective is needed to clarify heterogeneity in personality-related risk profiles.

**Figure 1:**
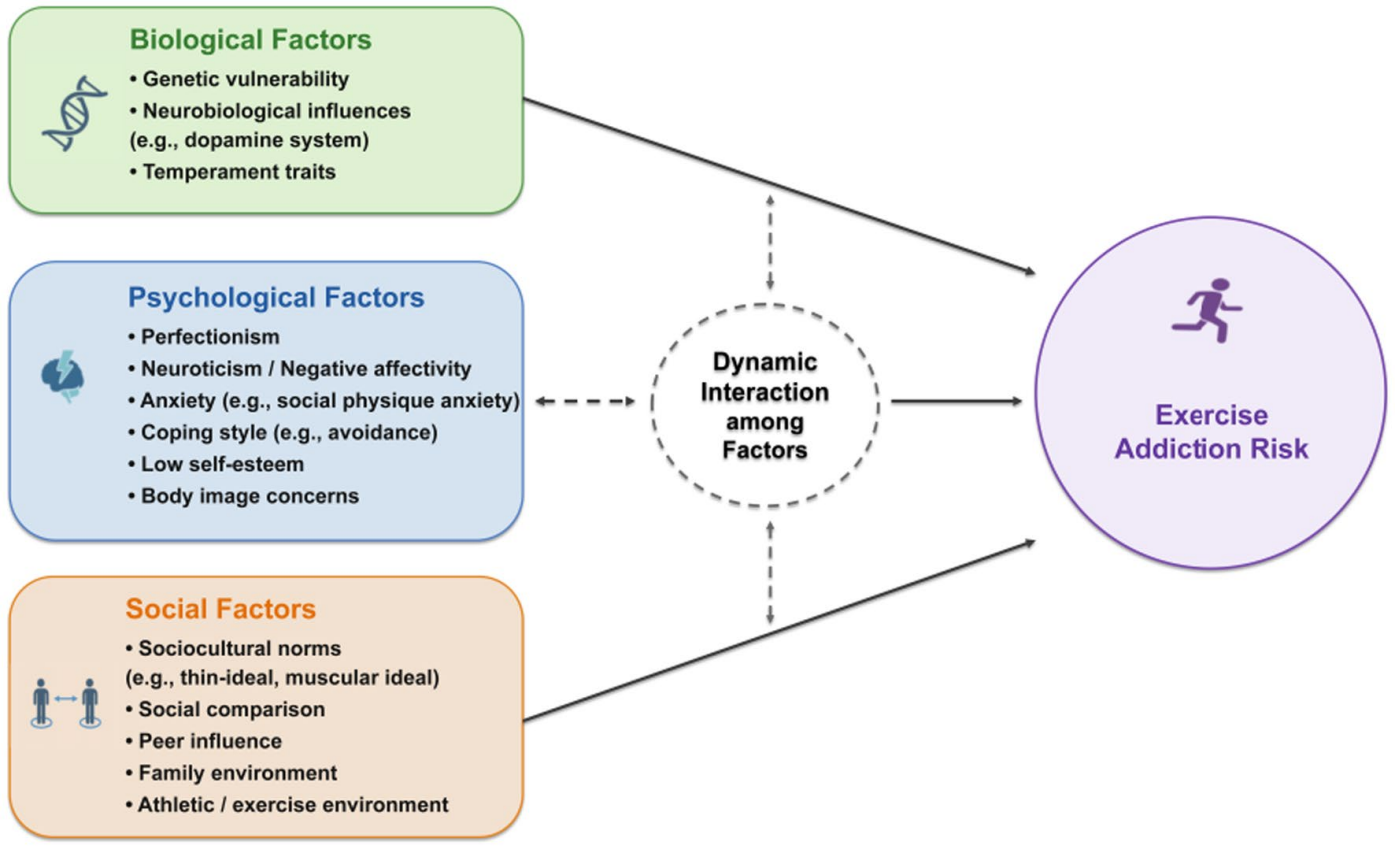
Biopsychosocial model of exercise addiction risk.

Perfectionism is one personality trait that has been repeatedly linked to EA. It is characterized by setting excessively high standards and engaging in critical self-evaluation (Frost et al., 1990). Clinical perfectionism has been associated with various psychological difficulties, including social anxiety, depression, eating disorders, and obsessive-compulsive symptoms (Egan et al., 2011). In the context of exercise, individuals with stronger perfectionistic tendencies may be more likely to maintain rigid exercise standards, compensate for missed exercise sessions, or continue training despite physical or psychological costs. Empirical studies have shown that individuals with higher EA scores tend to report higher levels of perfectionism (Grandi et al., 2011; Sallis et al., 2021), and the “personal standards” dimension of perfectionism has also been positively associated with EA-related symptoms (Taranis and Meyer, 2010).

Neuroticism is another important personality trait relevant to EA. It reflects a tendency to experience negative emotions, heightened sensitivity to stress, and lower tolerance of frustration (Birche et al., 2017). Individuals with higher neuroticism may use exercise as a coping strategy to escape or regulate negative emotions such as guilt, anxiety, irritability, or restlessness (Hausenblas and Giacobbi, 2004; Yoon and Barker Steege, 2013). Although exercise may initially function as an adaptive coping mechanism, it may become maladaptive when individuals increasingly depend on exercise to regulate emotional distress or avoid withdrawal-like feelings. In addition, classical and contemporary theories have suggested that perfectionism and neuroticism may overlap in maladaptive personality functioning. Adler (1938) regarded perfectionism as a neurotic form of overcompensation (Adler, 1938), while Hamachek (1978) emphasized that maladaptive perfectionism is often characterized by unrealistic standards and a strong need to avoid failure (Hamachek, 1978). Frost et al. (1993) also noted that perfectionism and neuroticism are closely associated with a range of psychological symptoms (Frost et al., 1993).

Despite this theoretical overlap, previous EA studies have generally examined perfectionism and neuroticism as separate predictors. This may obscure how the two traits co-occur within individuals and jointly contribute to EA risk. In other words, two individuals with similar levels of perfectionism may differ substantially in emotional vulnerability, and two individuals with similar neuroticism levels may differ in self-imposed standards and evaluative concerns. A variable-centered analysis may not fully capture these differences. Therefore, one important gap in the literature is the lack of person-centered evidence on whether distinct perfectionism–neuroticism profiles exist among university students and whether these profiles differ in EA levels.

In addition to personality traits, social evaluative concerns may also play an important role in EA. Social physique anxiety (SPA) refers to the anxiety individuals experience when they perceive that others may negatively evaluate their body shape or physical appearance (Hart et al., 1989). In contemporary fitness culture, body ideals and appearance-based comparisons may intensify concerns about physique and increase pressure to exercise for external approval. Previous studies have indicated that SPA is associated with excessive exercise behavior and exercise-related problems (Hurst et al., 2000; Zhu et al., 2024). Perfectionistic individuals may be particularly sensitive to perceived inadequacy in physical appearance, while individuals high in neuroticism may be more prone to anxiety and negative emotional responses in socially evaluative situations. Thus, SPA may provide a psychological pathway through which personality-related vulnerability is linked to EA. However, the mediating role of SPA has not been sufficiently examined within a personality-profile framework. Existing studies have provided evidence that perfectionism, neuroticism, and SPA are each associated with EA-related outcomes, but it remains unclear whether SPA statistically accounts for the association between latent personality-risk profiles and EA. This issue remains insufficiently addressed in the existing literature, particularly regarding the statistical pathway through which personality heterogeneity may be linked to EA. Clarifying this pathway may help explain why some students with higher personality-related vulnerability are more likely to report EA symptoms, especially when their exercise behavior is shaped by body-related social evaluation.

Latent profile analysis (LPA) offers a useful approach for addressing these gaps. LPA is a person-centered method that identifies unobserved subgroups within a population based on individuals’ response patterns across selected indicators (Oberski, 2016). Compared with traditional variable-centered methods, LPA can reveal whether specific combinations of perfectionism dimensions and neuroticism exist within the sample. This approach is particularly suitable for testing whether high perfectionism and high neuroticism co-occur in a subgroup of students and whether such a subgroup shows higher EA risk. Moreover, by combining LPA with mediation analysis, it is possible to examine whether SPA functions as a statistical mediator between personality-profile membership and EA.

**Figure 2:**
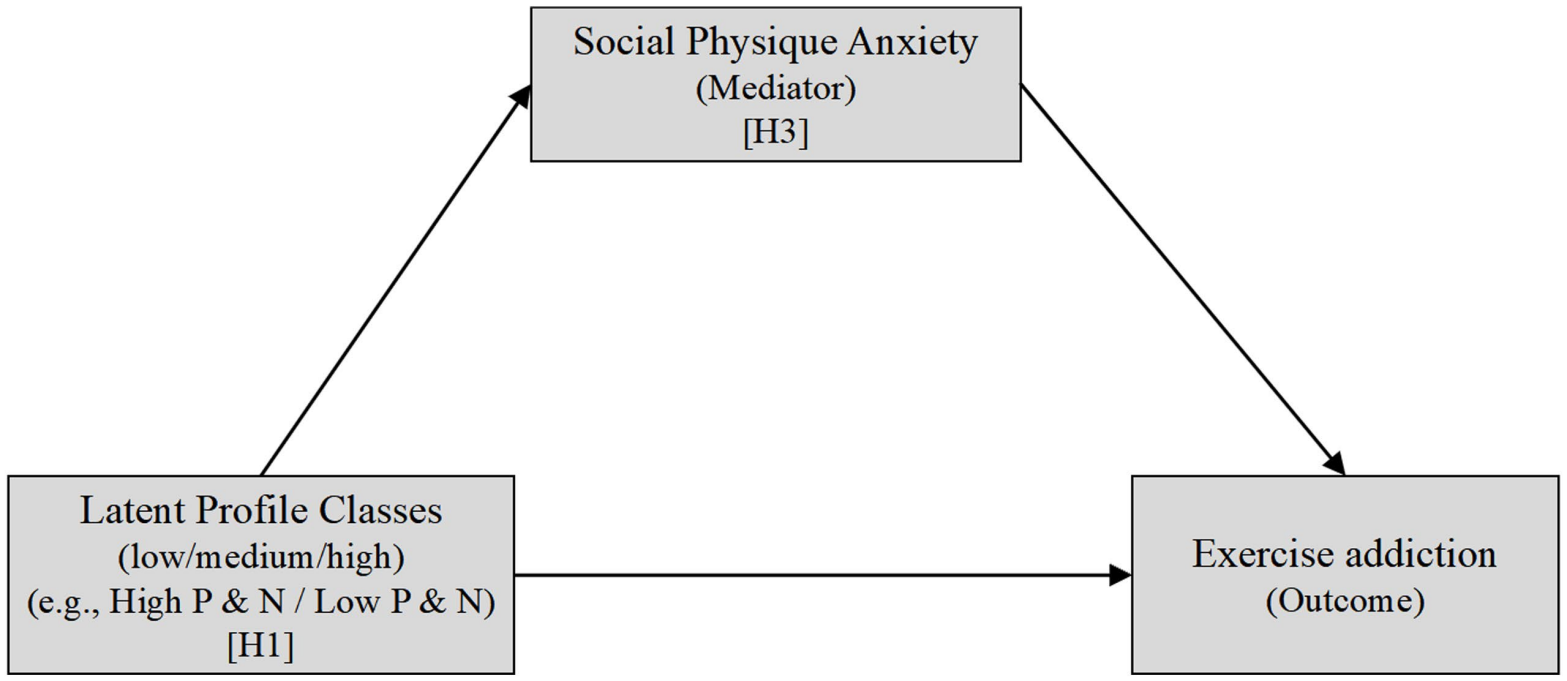
Diagram of theoretical models of perfectionism, neuroticism, SPA and EA. P & N, perfectionism and neuroticism.

Based on the results of previous studies and the above analysis, the following hypotheses are proposed: (1) among college students, distinct latent psychological profiles can be identified based on perfectionism dimensions and neuroticism scores (e.g., high-perfectionism/high-neuroticism type, low-perfectionism/low-neuroticism type). (2) Individuals belonging to different latent psychological profile classes exhibit significantly different levels of EA. (3) The mediating effect of SPA on the relationship between latent psychological profiles and EA varies across different profile subgroups (Figure 2).

## Method

### Design and participants

This study was conducted from April to May 2025 through the online questionnaires. Data were collected from two universities in each of Shandong, Henan, and Jiangsu provinces. Because the aim of this study was to examine EA-related risk among students with regular exercise participation, regular exercise participation was set as an intentional inclusion criterion. Inclusion criteria were as follows: (1) full-time college and vocational students aged between 18 and 26 years; (2) regular engagement in physical exercise, defined as participating in moderate-intensity or higher physical activity at least three times per week for a minimum of 30 min per session over the past 6 months; and (3) voluntary participation with signed informed consent. Exclusion criteria included: (1) currently undergoing psychological treatment or taking psychiatric medication; (2) presence of severe physical illnesses or motor impairments that interfere with regular exercise; and (3) having received structured psychological interventions or counseling within the past 3 months.

The study received approval from the Biomedical Research Ethics Committee of Henan University (HUSOM2025-614), and written informed consent was obtained from participants through the researchers at the respective universities. The informed consent form emphasized the strict protection of participant privacy and ensured the confidentiality of data. Participants were informed that they could withdraw from the study at any stage without providing a reason, and that withdrawal would not result in any negative consequences. All questionnaires were completed anonymously, with no personal information recorded or disclosed, ensuring the privacy and security of the participants.

### Sample size

In terms of sample size, the study followed the principle that the sample size should be 5 to 10 times the number of measurement items, with a minimum sample size of no less than 300 (DeVellis and Thorpe, 2021). To ensure the authenticity and accuracy of the data and to reduce the potential influence of common method variance, the study implemented the following procedural and quality control measures: (1) participation was voluntary, and participants were informed that their responses would be anonymous, confidential, and used only for research purposes; (2) all questionnaires were administered in quiet classroom settings under the supervision of academic or counseling staff to support independent and serious participation; (3) each participant was allowed to submit the questionnaire only once, with the online system programmed to prevent duplicate submissions; (4) reverse-scored items and consistency check items were included to detect random or inattentive responses; (5) questionnaires were excluded if they exhibited abnormally short completion times (e.g., less than 5 min) or contained logical inconsistencies. A total of 531 participants agreed to complete the survey. After excluding invalid responses based on the quality control criteria, 481 valid responses were retained, resulting in a valid response rate of 90.58%. Furthermore, considering the 481 valid samples, the required sample size for each subgroup, in the case of insufficient centroid separation, was approximately 30 (Dalmaijer, 2023). Therefore, the sample size for the three subgroups in this study was considered adequate and effective.

### Measurement

#### Perfectionism

The Frost Multidimensional Perfectionism Scale (FMPS) was developed by Frost et al. (1990) to measure the cognitive, emotional, and behavioral aspects of perfectionists. Zi and Zhou (2006) modified the scale to better suit Chinese culture, and conducted reliability and validity testing, resulting in the Chinese version of the FMPS. The scale consists of five dimensions: “Concern over Mistakes” (CM), “Organization” (O), “Personal Standards” (PS), “Parental Expectations” (PE), and “Doubts about Actions” (DA), with a total of 27 items. The scale uses a five-point Likert scale, where 1 represents “strongly disagree” and 5 represents “strongly agree.” In this study, the Cronbach’s *α* for the FMPS was 0.90, demonstrating good internal consistency.

#### Neuroticism

This study used the Neuroticism subscale of the 60-item Neo Five Factor Inventory-neuroticism (NEO-FFI-N) to assess the neuroticism level of participants (Costa Jr and McCrae, 1992). The Chinese version of the NEO-FFI-N has been validated in the Chinese population, showing good reliability and validity (Yang et al., 1999). The scale consists of 12 items, rated on a five-point Likert scale from 1 (“strongly disagree”) to 5 (“strongly agree”). Higher mean scores indicate higher neuroticism levels. In this study, the Cronbach’s *α* for the NEO-FFI-N was 0.64, indicating moderate internal consistency.

#### Social physique anxiety

The Social Physique Anxiety Scale (SPAS), developed by Xu (2003), was used in this study. This scale has been widely used in Chinese populations and has demonstrated good reliability and validity (Zhu et al., 2024). The scale includes three dimensions: “Fear of Negative Evaluation,” “Discomfort in Displaying Physical Appearance,” and “Social Comparison Anxiety,” with a total of 15 items. It uses a five-point Likert scale, where 1 represents “strongly disagree” and 5 represents “strongly agree.” Higher scores indicate greater social physique anxiety. In this study, the Cronbach’s *α* for the SPA was 0.90, indicating good internal consistency.

#### Exercise addiction

The Exercise Addiction Inventory (EAI) was used to assess participants’ symptoms and risks of EA (Griffiths et al., 2005). This scale is a brief screening tool consisting of six items. The Chinese version of the EAI has been validated in the Chinese population, showing good reliability and validity (Li et al., 2015). The questionnaire uses a five-point Likert scale, with scores ranging from 1 (“strongly disagree”) to 5 (“strongly agree”). The final evaluation is based on the total score of all items. A total score of 24 or greater is considered to indicate the presence of EA symptoms and risks. In this study, the Cronbach’s α for the EAI was 0.91, indicating good internal consistency.

### Data analysis

**Figure 3:**
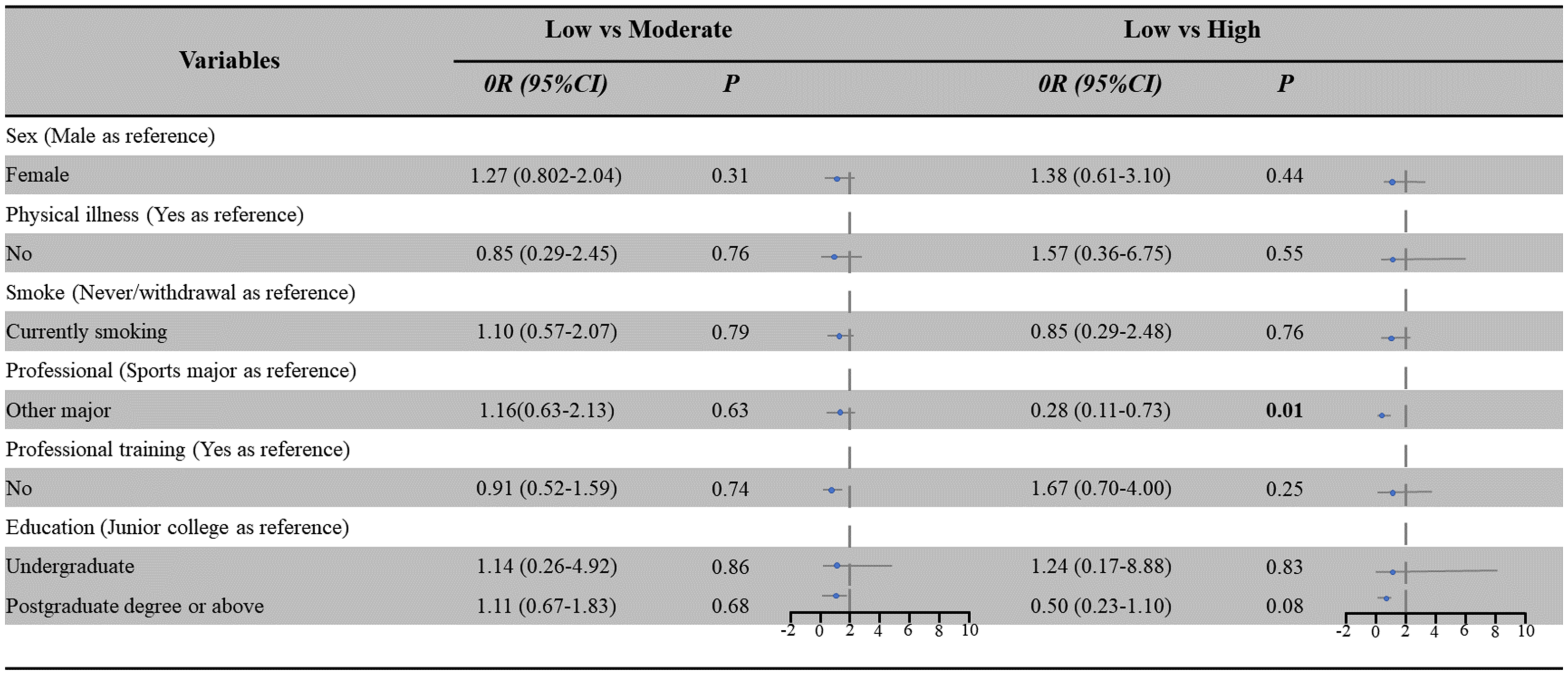
Logistic regression results.

**Figure 4:**
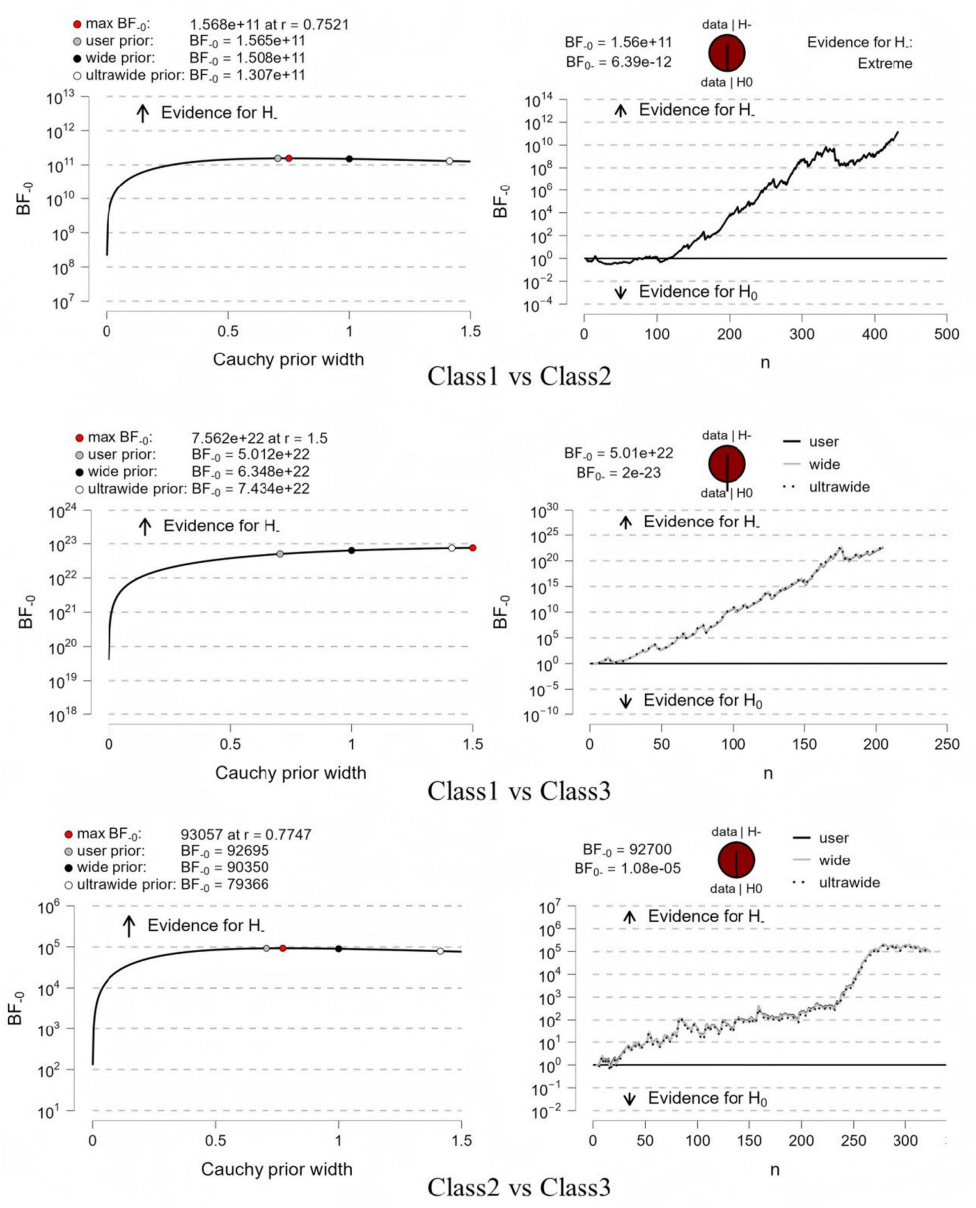
Bayes factor robustness and sequential based on latent profile analysis.

We first conducted descriptive statistical analyses of participants’ demographic characteristics using SPSS 26.0. Pearson correlation analyses were then performed to examine linear relationships among perfectionism, neuroticism, SPA, and EA. To identify potential latent subgroups characterized by distinct levels of perfectionism and neuroticism, we employed LPA using Mplus 8.0, with model fit indices serving as evaluation criteria. In the LPA, profile indicators consisted of the five FMPS subscale scores and the NEO-FFI-N neuroticism score, all calculated as item-level mean scores on a 1–5 scale. Univariate regression analyses were subsequently conducted to explore factors associated with the LPA-based profiles, and results were visualized using forest plots (see Figure 3). Next, Bayesian independent samples *t*-tests (see Figure 4) were conducted in JASP to compare EAI scores across subgroups. Finally, mediation analysis was performed in SPSS 26.0 using PROCESS (Model 4) to examine whether SPA showed a statistical mediating role in the association between personality subgroups and EA. Relevant demographic variables were included as covariates in the mediation models to reduce the potential influence of demographic composition differences across latent profiles.

### Research results

#### Demographic variables

Among all participants, 235 were male, accounting for 48.9% of the total, and 246 were female, accounting for 51.1%. The participants’ ages ranged from 18 to 26 years, with an average age of 21.31 years (*SD* = 2.37). The average BMI was 21.59 ± 3.88, with most students falling within the normal BMI range. Of the participants, 140 were students majoring in physical education, accounting for 29.1%, while 341 were from other majors, accounting for 70.9%. Regarding educational level, 357 were undergraduates, accounting for 74.2%; 113 were graduate students or above, accounting for 23.5%; and 11 were vocational students, accounting for 2.3%. Detailed information is shown in Table 1.

**Table 1:** Demographic characteristics of the sample (*N* = 481).

Characteristics	*M* ± SD	Shapiro–Wilk test (*p*-value)	*N*	%
Age (year)	21.31 ± 2.37		-	-
BMI	21.59 ± 3.88			
Sex				
Females			246	51.1
Males			235	48.9
Physical illness				
Yes			18	3.7
No			463	96.3
Smoke				
Current smoking			60	12.5
Never/quit			421	87.5
Professional				
Sports major			140	29.1
Other majors			341	70.9
Athletic training experience				
Yes			253	52.6
No			228	47.4
Education				
Junior college			11	2.3
Undergraduate			357	74.2
Postgraduate degree or above			113	23.5
Perfectionism	2.84 ± 0.56	<0.001		
CM	1.90 ± 0.86	<0.001		
PS	2.94 ± 0.73	<0.001		
PE	2.83 ± 0.90	<0.001		
DA	2.77 ± 0.93	<0.001		
O	3.73 ± 0.75	<0.001		
Neuroticism	2.75 ± 0.49	<0.001		
Exercise addiction	2.89 ± 1.09	<0.001		
Social physique anxiety	3.04 ± 0.88	<0.001		
FNE	3.00 ± 0.92	<0.001		
DSP	3.10 ± 0.91	<0.001		
ASC	3.03 ± 1.02	<0.001		

CM, concern over mistakes; PS, personal standards; PE, parents’ expectations; DA, doubts about actions; O, organization; FNE, fear of negative evaluation; DSP, distress in social performance; ASC, anxiety about social comparison.

#### Descriptive statistics and correlation analysis

**Figure 5:**
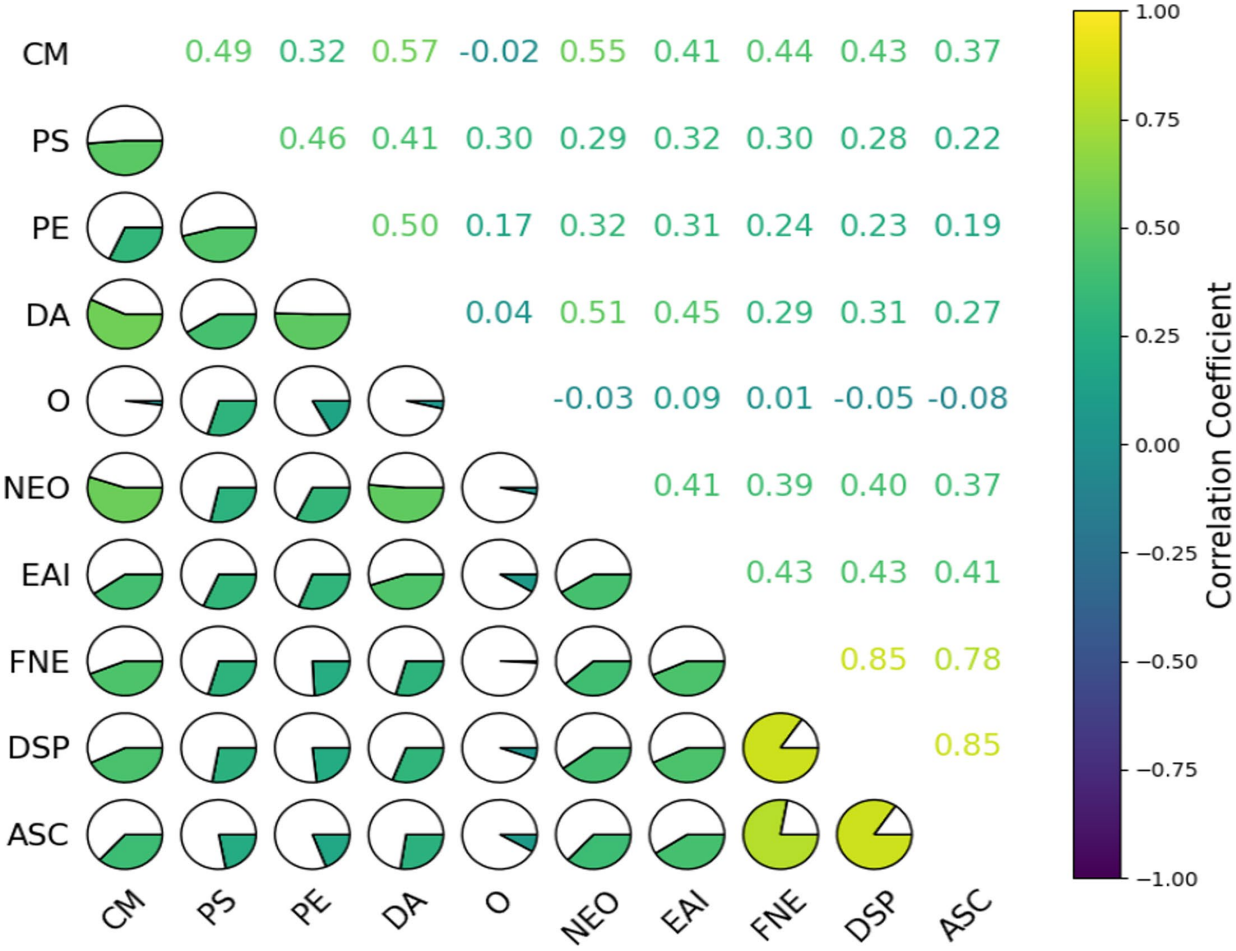
Pearson correlation heatmap. CM, concern over mistakes; PS, personal standards; PE, Parents’ expectations; DA, doubts about actions; O, organization; NEO, neuroticism; EA, exercise addiction; FNE, fear of negative evaluation; DSP, distress in social performance; ASC, anxiety about social comparison.

The results showed that the variance explained by the first factor was 17.7%, which was below the commonly used threshold of 40%. Together with the procedural controls described above, this result suggested that common method bias was unlikely to be a serious concern in the present study (Podsakoff et al., 2003). Perfectionism was positively correlated with EAI scores (*r* = 0.46, *p* < 0.001), as well as with neuroticism (*r* = 0.49, *p* < 0.001) and SPA (*r* = 0.38, *p* < 0.001). Additionally, neuroticism was positively correlated with both SPA (*r* = 0.41, *p* < 0.001) and EA (*r* = 0.41, *p* < 0.001). SPA was also positively correlated with EA (*r* = 0.45, *p* < 0.001). Figure 5 illustrates the Pearson correlation heatmap.

#### Latent profile analysis of perfectionism and neuroticism

Table 2 summarizes the fit indices of different category models (Class 1 – Class 5) in the LPA. As the model complexity increased, the AIC, BIC, and ABIC values generally decreased, suggesting that introducing more categories improved the model’s fit to some extent. Specifically, the three-category model achieved relatively lower values of AIC (6003.07), BIC (6111.65), and ABIC (6029.12), indicating its relative advantage in fitting performance. From the statistical significance tests for model comparison, the Bootstrap Likelihood Ratio Test (BLRT) was highly significant (*p* < 0.001) for all models, supporting the inclusion of each latent class. Regarding classification accuracy, the entropy value for the three-category model was 0.80, which was better than the two-category model (0.75) and close to the four-category model (0.81), demonstrating good class separability. Although the information criteria continued to decrease in the four- and five-class models, the LMR test was no longer significant for these models, indicating that additional classes did not provide a statistically clear improvement over the three-class solution. Beyond model parsimony, the three-class model was selected because it yielded a clear and theoretically meaningful low–moderate–high pattern of perfectionism–neuroticism profiles. This profile structure was consistent with the purpose of identifying personality-related risk gradients and allowed for more interpretable comparisons of EAI scores and mediation pathways. In contrast, the four- and five-class solutions did not yield additional substantively distinct profiles and appeared to further subdivide existing risk levels. Therefore, considering statistical fit, classification quality, class size, and theoretical interpretability, the three-class model was retained.

**Table 2:** Potential profiling of perfectionism and neuroticism.

Indicators	LPA model				
	Class 1	Class 2	Class 3	Class 4	Class 5
Fit statistics					
AIC	6632.88	6202.80	**6003.07**	5929.07	5865.01
BIC	6682.99	6282.14	**6111.65**	6066.88	6032.04
ABIC	6652.99	6221.84	**6029.12**	5962.14	5905.09
LMR(P)	–	0.06	**0.02**	0.06	0.25
BLRT	–	< 0.001	**<0.001**	< 0.001	< 0.001
Entropy	–	0.75	**0.80**	0.81	0.78
K	12	19	**26**	33	40
C1	481 (100%)	329 (68.40%)	**157 (32.64%)**	259 (53.85%)	33(6.86%)
C2	–	152 (31.60%)	**276 (57.38%)**	145 (30.15%)	116 (24.11%)
C3	–	–	**48 (9.98%)**	33 (6.86%)	206 (42.83%)
C4	–	–	**–**	44 (9.14%)	105 (21.83%)
C5	–	–	**–**	–	21 (4.37%)

Bold values indicate the best-fitting model selection indices and the final selected latent profile solution.

**Figure 6:**
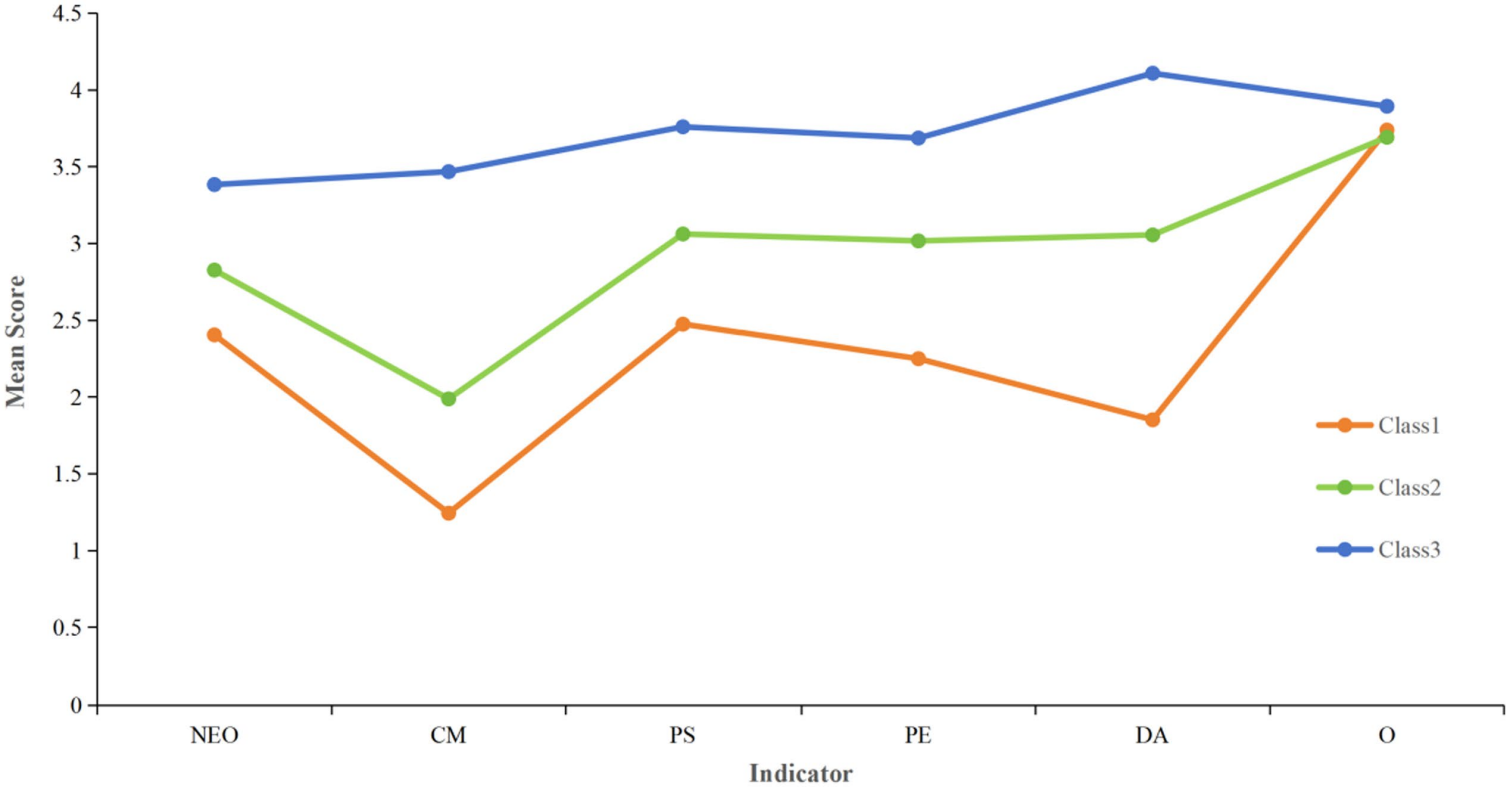
Latent profile analysis model. NEO, neuroticism; CM, concern over mistakes; PS, personal standards; PE, parents’ expectations; DA, doubts about actions; O, organization.

Figure 6 displays the three-profile model based on Latent Profile Analysis (LPA). In terms of sample distribution, the three-category model consisted of: Class 1 with 157 participants (32.64% of the total sample), Class 2 with 276 participants (57.38%), and Class 3 with 48 participants (9.98%). Based on the class-specific mean-score patterns of the six LPA indicators (Supplementary Table S1), participants were classified into three subgroups: the low perfectionism–neuroticism profile, moderate perfectionism–neuroticism profile, and high perfectionism–neuroticism profile.

Moreover, multivariate regression analysis showed that academic major, particularly being a sports major, significantly distinguished Class 1 from Class 3. Figure 3 illustrates the logistic regression results based on latent traits. To ensure the stability of model interpretation, this study used a screening criterion that the proportion of each latent class should not be below 5% (Tein et al., 2013). Considering the fit indices, statistical significance, classification quality, and sample distribution, the three-class latent model was found to best match the data structure of this study. Subsequent analyses were based on this model.

To further examine demographic correlates of latent profile membership, logistic regression analyses were conducted using the low perfectionism–neuroticism profile as the reference group. Figure 3 presents the odds ratios and 95% confidence intervals for the comparisons between the low and moderate profiles and between the low and high profiles. In this forest plot, odds ratios greater than 1 indicate a higher likelihood of belonging to the comparison profile, whereas odds ratios less than 1 indicate a lower likelihood relative to the reference profile.

As shown in Figure 3, most demographic variables, including sex, physical illness, smoking status, athletic training experience, and educational level, did not significantly distinguish the latent profiles. However, academic major showed a significant difference in the comparison between the low and high profiles. Specifically, compared with students majoring in physical education, students from other majors were less likely to belong to the high perfectionism–neuroticism profile. This suggests that students with sports-related academic backgrounds may be more represented in the high-risk personality profile.

#### Mediation analysis based on latent profiles

Based on the three categories derived from the latent profiles (see Figure 6), We conducted cross-sectional statistical mediation analyses in turn, controlling for relevant demographic variables, such as sex, academic major, and other background characteristics. Using the low perfectionism–neuroticism profile as the reference class, the mediation model for the moderate perfectionism–neuroticism profile showed that the total effect of Class 2 on EA scores was significant (Effect = 0.78, 95% CI [0.59, 0.97]). Furthermore, the direct effect (Effect = 0.54, 95% CI [0.35, 0.73]) and indirect effect (Effect = 0.24, 95% CI [0.16, 0.34]) were also significant, suggesting that the results were consistent with a partial statistical mediating role of social physique anxiety in the association between the moderate perfectionism–neuroticism profile and EA scores. Similarly, using the low perfectionism–neuroticism profile as the reference class, the mediation model for the high perfectionism–neuroticism profile showed that the total effect was significant (Effect = 1.60, 95% CI [1.28, 1.92]). The direct effect (Effect = 1.18, 95% CI [0.86, 1.50]) and indirect effect (Effect = 0.43, 95% CI [0.28, 0.60]) were also significant, and the results were consistent with a partial statistical mediating role of SPA in the association between the high perfectionism–neuroticism profile and EA. Specific values can be found in Tables 3, 4.

**Table 3:** Unstandardized regression coefficients for the mediation models.

Variables	*β*	*SE*	*t*	*p*	Bootstrap 95%CI		*R^2^*
					Lower	Upper	
Outcome variable: SPA							0.18
Class 2	0.61	0.08	7.56	<0.001	0.45	0.77	
Class 3	1.07	0.13	7.99	<0.001	0.81	1.33	
Outcome variable: EAI							0.30
Class 2	0.54	0.10	5.49	<0.001	0.35	0.73	
Class 3	1.18	0.16	7.18	<0.001	0.86	1.50	
SPA	0.40	0.05	7.52	<0.001	0.29	0.50	

**Table 4:** Mediating effects and effect sizes.

Effect type	Grouping variable	Effect	*SE*	Bootstrap 95%CI	
				Lower	Upper
Indirect effect	Class 2	0.24	0.04	0.16	0.34
	Class 3	0.43	0.08	0.28	0.60
Direct effect	Class 2	0.54	0.10	0.35	0.73
	Class 3	1.18	0.16	0.86	1.50
Total effect	Class 2	0.78	0.10	0.59	0.97
	Class 3	1.60	0.16	1.28	1.92

Bayesian independent-samples t-tests were further conducted to compare EA levels across the three latent profiles. Figure 4 presents both Bayes factor robustness analyses and sequential analyses for the pairwise comparisons among the profiles. The robustness plots show whether the Bayes factors remain stable across different prior widths, whereas the sequential plots illustrate how the evidence changes as the sample size increases. As shown in Figure 4, the Bayes factors for the comparisons between Class 1 and Class 2, Class 1 and Class 3, and Class 2 and Class 3 all provided strong to extreme evidence in favor of differences in EA levels across profiles. The sequential analyses further indicated that the evidence for group differences increased progressively with the accumulation of observations and remained consistently in favor of the alternative hypothesis. These findings support the conclusion that EAI scores differed significantly across the latent perfectionism–neuroticism profiles, with higher-risk profiles showing higher EAI scores levels.

## Discussion

This study identified three perfectionism–neuroticism profiles among university students and found that these profiles were differentially associated with EA scores. Compared with the low perfectionism–neuroticism profile, the moderate and high profiles showed higher levels of EA, and SPA partially mediated these associations. These findings are consistent with the interactional model of EA, which emphasizes that EA may emerge from the combined influence of individual psychological vulnerability and social-contextual factors (Egorov and Szabo, 2013; McNamara and McCabe, 2012). In this study, perfectionism–neuroticism profiles reflected personality-related vulnerability, whereas SPA represented body-related social evaluative concerns. Together, these results suggest that EA among university students should not be understood solely as excessive exercise behavior, but rather as a psycho-behavioral pattern involving both personality traits and social evaluation processes.

The LPA results revealed a graded pattern of personality-related vulnerability. The high perfectionism–neuroticism profile was characterized by relatively higher levels of perfectionistic tendencies and neuroticism, reflecting the co-occurrence of rigid self-standards and emotional vulnerability. The moderate profile showed an intermediate pattern, whereas the low profile served as a relatively low-risk reference group. This profile structure supports the value of a person-centered approach. LPA allows researchers to identify unobserved subgroups based on individuals’ response patterns across selected indicators, thereby capturing heterogeneity that may be obscured in traditional variable-centered analyses (Oberski, 2016). Rather than treating perfectionism and neuroticism as isolated predictors, the present findings suggest that their configuration within individuals may provide a more nuanced understanding of EA-related risk.

These results also extend previous theoretical views on the overlap between perfectionism and neuroticism. Perfectionism is characterized by excessively high standards and critical self-evaluation (Frost et al., 1990), whereas neuroticism reflects a tendency toward negative emotionality, stress sensitivity, and low frustration tolerance (Birche et al., 2017). Earlier theoretical perspectives have suggested that maladaptive perfectionism is closely related to neurotic functioning, particularly through unrealistic standards, fear of failure, and heightened sensitivity to negative evaluation (Adler, 1938; Frost et al., 1993; Hamachek, 1978b). The present study adds to this literature by showing that perfectionism and neuroticism can form distinct latent profiles among university students and that these profiles are associated with different levels of EA. This finding indicates that EA may be more strongly linked to personality configurations involving both self-critical standards and emotional instability than to either trait considered separately.

The mediation results further suggest that SPA may represent a body-related social evaluative pathway linking personality-risk profiles to EA. SPA refers to anxiety arising from perceived negative evaluation of one’s body shape or physical appearance (Hart et al., 1989). Previous studies have shown that SPA is associated with exercise-related problems and exercise dependence symptoms (Hurst et al., 2000; Zhu et al., 2024). From a social evaluative perspective, individuals who internalize unrealistic body ideals or experience heightened appearance-related concerns may be more likely to engage in exercise for external approval or anxiety regulation (Leary and Kowalski, 1990). In the present study, students in the high perfectionism–neuroticism profile may have been more likely to experience body-related evaluative concerns when they perceived themselves as failing to meet self-imposed or socially valued standards. In this context, exercise may function not only as a health-promoting behavior but also as a strategy for managing body-related anxiety and social evaluative pressure.

The indirect association through SPA was also observed in the moderate perfectionism–neuroticism profile, although the effect was weaker than that of the high profile. This suggests that SPA may not be limited to individuals with the highest personality-related vulnerability, but may operate along a continuum of risk. In contrast, the low perfectionism–neuroticism profile showed the lowest levels of both SPA and EA, supporting its role as a low-risk reference group. Overall, these findings suggest that the relationship between personality profiles and EA is partly explained by body-related social evaluative concerns. However, given the cross-sectional design of this study, this indirect association should be interpreted as a statistical mediation rather than evidence of a causal mechanism.

These findings have practical implications for the prevention of EA-related symptoms and risk in university settings. Screening and intervention efforts may benefit from considering both personality-risk profiles and SPA, rather than focusing only on exercise frequency or behavioral symptoms. For students with higher perfectionism–neuroticism profiles, interventions may need to address emotional regulation, self-critical standards, and body-related social evaluative concerns. For students with moderate risk, structured behavioral guidance and support for healthy exercise motivation may be useful. Such profile-informed approaches may help promote more stable and sustainable physical activity patterns among university students.

### Limitations and practical implications

This study has several limitations. First, this study did not aim to compare the proportion of physically inactive students with the proportion of students at risk of EA in the general university population. Because the sample was restricted to students with regular exercise participation, the findings should be interpreted as applying primarily to students who regularly engage in exercise rather than to all university students. Future studies should conduct broader screening in general university samples to examine both physical inactivity and EA-related risk. Second, due to the cross-sectional design, causal relationships cannot be inferred. In future research, we recommend conducting longitudinal follow-up studies of the subgroups identified through LPA over a longer time span to examine differences in the developmental trajectories of EA across latent classes, as well as changes in each subgroup’s response to interventions, risk outcomes, and other evolving patterns. Third, this study only examined the roles of perfectionism and neuroticism. Although these variables explained a meaningful portion of the variance in EA, other important factors, such as self-esteem and extraversion, may also play a role. Future studies should further explore the impact of additional personality traits on EA. Finally, the NEO-FFI-N showed only modest internal consistency in the present sample (Cronbach’s *α* = 0.64), so findings related to neuroticism should be interpreted with caution. Future studies should further validate this measure in similar university student samples.

Given the findings of this study, early prevention and intervention are crucial in addressing EA among university students. We recommend that colleges and universities incorporate brief yet psychometrically sound psychological assessment tools—such as the FMPS or the SPA —into mental health screenings or health education courses. Given the distinct psychological risks identified across latent profiles, targeted intervention efforts should prioritize the high perfectionism–neuroticism profile. Individuals in this subgroup are particularly vulnerable to maladaptive exercise behaviors associated with body dissatisfaction and social evaluation. Interventions should emphasize the emotional and health-related benefits of physical activity, rather than performance- or appearance-based goals. Complementary strategies such as body image acceptance training and emotional regulation support may help shift their motivation from extrinsic pressure to intrinsic wellness. For the moderate perfectionism–neuroticism profile, reinforcing behavioral consistency through structured plans and social support is recommended to sustain healthy habits. The low perfectionism–neuroticism profile, while low-risk, can be mobilized as peer role models to foster a positive and health-promoting exercise culture. Additionally, altering exercise routines may help reduce EA symptoms. For example, individuals at risk of EA may switch to swimming; alternating between endurance and strength training, or transitioning from individual to group exercises to increase social interaction, may also be beneficial.

## Conclusion

The following conclusions were drawn from the study: (1) based on perfectionism and neuroticism scores, college students can be classified into three latent subgroups: high, moderate, and low perfectionism–neuroticism profiles; (2) the three subgroups were significantly associated with EAI scores; (3) SPA showed a statistical mediating role in the association between latent profile membership and EAI scores.

## Data Availability

The raw data supporting the conclusions of this article will be made available by the authors, without undue reservation.
