# Supplementary material for: The relationship between perfectionism, neuroticism, and exercise addiction risk: a latent profile analysis and the mediating role of social physique anxiety
Source: Front Psychol. 2026 Jun 26;17:1857926. doi: 10.3389/fpsyg.2026.1857926 (PMC13350270; doi:10.3389/fpsyg.2026.1857926)
Supplement: Supplementary file 1 [file Table_1.DOCX]

**Table S1 Class-specific mean scores of the six LPA indicators**

| Indicator | Class 1-Low P–N profile  （*N* = 157） | Class 2-Moderate P–N profile  （*N* = 276） | Class 3-High P–N profile  （*N* = 48） |
| --- | --- | --- | --- |
| NEO | 2.405 | 2.827 | 3.384 |
| CM | 1.244 | 1.988 | 3.468 |
| PS | 2.475 | 3.062 | 3.76 |
| PE | 2.25 | 3.017 | 3.687 |
| DA | 1.852 | 3.056 | 4.109 |
| O | 3.739 | 3.692 | 3.894 |

Note. Values are item mean scores on the original 1–5 scale. P–N = perfectionism–neuroticism; NEO = neuroticism; CM = concern over mistakes; PS = personal standards; PE = parental expectations; DA = doubts about actions; O = organization.
